# Physical Education

**Published:** 1861-12

**Authors:** 


**PHYSICAL EDUCATION.**—Dr. Lewis's Normal Institute, for Physical Education, will open its second course on January 2d, 1862, at 20 Essex street, Boston, where circulars can be had.

